# Design and experimentation of multi-fruit envelope-cutting kiwifruit picking robot

**DOI:** 10.3389/fpls.2024.1338050

**Published:** 2024-02-05

**Authors:** Min Fu, Shike Guo, Anyu Chen, Renxuan Cheng, Xiaoman Cui

**Affiliations:** College of Mechanical and Electrical Engineering, Northeast Forestry University, Harbin, China

**Keywords:** kiwifruit, picking robot, multi-fruits envelope-cutting type, kinematic analysis, trajectory planning

## Abstract

Currently kiwifruit picking process mainly leverages manual labor, which has low productivity and high labor intensity, meanwhile, the existing kiwifruit picking machinery also has low picking efficiency and easily damages fruits. In this regard, a kiwifruit picking robot suitable for orchard operations was developed in this paper for kiwifruit grown in orchard trellis style. First, based on the analysis of kiwifruit growth pattern and cultivation parameters, the expected design requirements and objectives of a kiwifruit picking robot were proposed, and the expected workflow of the robot in the kiwifruit orchard environment was given, which in turn led to a multi-fruit envelope-cutting kiwifruit picking robot was designed. Then, the D-H method was used to establish the kinematic Equations of the kiwifruit-picking robot, the forward and inverse kinematic calculations were carried out, and the Monte Carlo method was used to analyze the workspace of the robot. By planning the trajectory of the robotic arm and calculating critical nodes in the picking path, the scheme of trajectory planning of the robot was given, and MATLAB software was applied to simulate the motion trajectory as well as to verify the feasibility of the trajectory planning scheme and the picking strategy. Finally, a kiwifruit picking test bed was set up to conduct picking tests in the form of fruit clusters. The results show that the average time to pick each cluster of fruit was 9.7s, the picking success rate was 88.0%, and the picking damage rate was 7.3%. All the indicators met the requirements of the expected design of the kiwifruit-picking robot.

## 1 Introduction

Kiwifruit is highly nutritious, known as the “king of fruit”, and has significant market demand. China is the world’s largest agricultural country in terms of kiwifruit planting area and production, with its annual yield exceeding 3 million tons (Fazayeli et al., 2019). In order to timely, efficiently, and carefully harvest kiwifruit, annual orchards require a large seasonal manual labor for harvesting, with labor costs accounting for more than 25% of annual production costs (García-Quiroga et al., 2015). With the scale-up of the kiwifruit planting industry, the traditional manual picking method is difficult to meet the current market demand. Faced with the increasing labor shortage, the development of efficient and adaptable kiwifruit harvesting robots is of great practical value.

Fruit and vegetable-picking robots are generally composed of robotic arm, end-effector, mobile mechanism, and control system. In recent years, scholars have conducted a series of studies on fruit and vegetable picking robots such as kiwifruit (Fang et al., 2023; Gao et al., 2023), apple (Miao and Zheng, 2019; Kuznetsova et al., 2020), citrus (Mehta and Burks, 2014; Sun et al., 2023), tomato (Ling et al., 2019; Wang T. et al., 2023) and so on. For example, Williams et al. (2020) designed a four-armed parallel kiwifruit-picking robot, which separated the fruit from the stalk by rotation after the end-effector gripped the kiwifruit with two fingers, the fruit was automatically collected into a fruit box after each picking action the average time required for harvesting each fruit was 5.5 seconds, with a fruit recognition rate of 76.3% and a successful harvesting rate of 51%. However, the robot had a problem that utilized two fingers to hold the fruit process, which was easy to affect the neighboring fruits. Arad et al. (2020) designed a six-degrees-of-freedom robotic arm bell pepper picking robot, after obtaining the position of the bell pepper with an RGB-D camera, the end-effector grasped the bell pepper and cut off the fruit stalk by using a vibrating cutter. Subsequently, the picked fruit was sent by the robotic arm to the designated collection box, and the average time required for harvesting each fruit was 24 seconds, with a harvesting success rate of up to 61%. Since the robot didn’t have an automatic collection device, it resulted in it taking up half of the overall harvest time in collecting the fruit. Rong et al. (2022) designed a watermelon harvesting robot that employed the improved YOLOv5s-CBAM algorithm for watermelon identification and localization. After the detection system recognized the watermelon, the control system drove four flexible fingers to clamp the watermelon, whose harvesting success rate was 93.3%. However, the robot’s detection system was susceptible to the effects of strong illumination and occlusion, and the harvesting success rate would be significantly reduced when the watermelon was partially shaded by leaves.

Improving fruit-picking efficiency and ensuring non-destructive fruit picking are two key criteria of fruit-picking robots, in which non-destructive picking is mainly ensured by the end-effector (Fu et al., 2015). For example, Mu et al. (2020) designed a wrapped kiwifruit-picking end-effector that used two bionic fingers to wrap the fruit before repeatedly bending the fingers to separate the fruit from the stalk. Silwal et al. (2017) developed a clamped apple-picking end-effector consisting of three actuators and three fingers, where after the fruit was clamped by the three fingers, the fruit was rotated by the base until the fruit was separated from the stalk. However, the end-effector was not equipped with a pressure sensor, which made it easy to damage the fruit when the clamping force was too large. And Hohimer et al. (2019) designed a 3-finger pneumatic gripping soft end-effector that could control picking pressure by adjusting the input air pressure, preventing fruit damage, though with lower picking efficiency. Wang et al. (2018) designed a bite-type citrus picking end-effector by simulating the biting action of a snake’s mouth, which could cut off the fruit stalk while gripping the fruit, However, there was a risk of fruit damage if the clamping was not precise. Xu L. et al. (2018) designed a suction-type navel orange picking end-effector, which used a suction cup to hold the fruit and then non-destructively separated the fruit from the stalk using a cutter. However, during fruit capture, it was prone to sucking debris, such as branches and leaves around the fruit, into the end-effector.

The above studies provide valuable insights for the development of harvesting techniques for kiwifruit and other fruits and vegetables. However, the current kiwifruit-picking end-effector is mostly of a single-fruit clamping or sucking structure, which is susceptible to two problems during the fruit-picking process: A clamping or sucking method of kiwifruit is unstable when gripping the fruit, leading to potential fruit loss. Approximately 87% of kiwifruit are distributed in clusters within the canopies (Fu et al., 2019), and the use of single-fruit picking has a high likelihood of impacting neighboring fruits, thereby leading to fruit drop. Given the above analysis, a kiwifruit picking robot was designed for kiwifruit grown in orchard trellis type, which could realize the continuous picking of multiple fruits by adopting the operation mode of enveloping the fruits - cutting off the fruit stalks - and collecting the fruits in pipelines; Then, MATLAB software was used to perform forward and reverse kinematics analysis of the kiwifruit picking robot, and the workspace range of the robot was solved. By planning the trajectory of the robotic arm and calculating the critical nodes in the picking path, a scheme of the robot picking strategy was given. Finally, a kiwifruit-picking test bed was set up to verify the performance of the kiwifruit-picking end-effector.

## 2 Design requirements

### 2.1 Kiwifruit cultivation parameters

**Figure 1:**
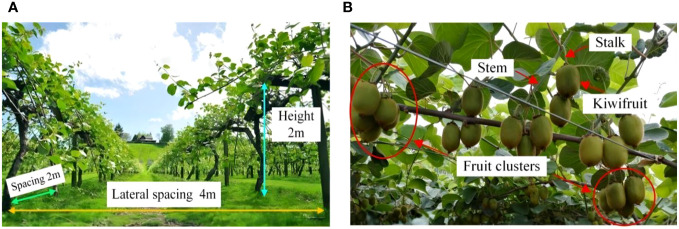
Kiwifruit orchard environment: **(A)** parameters of the trellis, **(B)** distribution of kiwifruit on the trellis. **(A)** Basic parameters of the kiwifruit.

In this study, the kiwifruit planted in trellis style at the kiwifruit industry base in Mei Xian County, Baoji City, Shanxi Province was used as the research object, and the kiwifruit orchard planting scenarios were characterized as follows. ① The trellis for kiwifruit cultivation is mainly made of steel tubes and steel wires, with supporting steel tubes between the trunks, and steel wires at the top of the tubes to fix the extended branches, so that the fruits grow naturally drooping. ② Orchard trellises have an average lateral spacing of 4m, a longitudinal spacing of 2m, and a height of 2m. ③ The fruits are generally distributed in clusters in a spatial range of 1.5~1.8m in height from the ground, and there is generally 3~7 fruits in each cluster, as shown in **Figure 1**.

In this paper, 50 ripened brown kiwifruits produced in Mei Xian County were randomly selected, and their physical parameters were measured. Electronic vernier calipers (with a precision of 0.01 mm) were used to measure the length, width, and thickness of the fruits, and the length of the fruit stalk. The weight of the fruits was measured using electronic scales (with a precision of 0.1 g). The data are presented in **Table 1**.

**Table 1:** Basic parameters of the kiwifruit.

Parameter	Quality/g	Length/mm	Width/mm	Thickness/mm	Fruit stalk length/mm
Sample	82.3	61.4	49.5	43.4	45.5
	92.6	67.5	53.3	50.6	52.2
	115.8	72.7	56.7	48.3	68.6
	……	……	……	……	……
Minimum value	82.3	61.4	49.5	43.4	45.5
Maximum value	115.8	72.7	56.7	48.3	68.6
Range	33.5	11.3	7.2	4.9	23.1
Average value	96.9	67.2	53.2	47.4	55.3

B. Parameters of fruit stalk separation.

When the kiwifruit is mature, the fruit stalk and the fruit form a separation layer with vascular bundles as the main tissue structure. Vascular bundles are tubular hollow fibrous tissue that can withstand strong tensile stresses but not easily withstand shear forces. The main cause of fruit abscission is the shear force on the separating layer of the fruiting stalk, which causes the vascular bundles to be fractured. In this regard, if a mechanical device is used to grab the kiwifruits and then a cutter is used to cut off the fruit stalks, the fruits can be picked without damage.

From the study in reference (Fu et al., 2015), it could be observed that when the fruit stalk formed a 180° angle with the inertia axis of the fruit, the maximum force required to separate the fruit stalk was 12.8 N. When the angle was 90°, the force required for separation was 1.7 N, and at a 60° angle, the minimum force needed to separate the fruit stalk was 1.3 N. The reasons were analyzed as follows: when the clamp angle was 180°, the vascular bundles were mainly subjected to tensile stress, and the separating force of the fruit stalk was larger, and when the clamp angle was 90° and 60°, the vascular bundles were mainly subjected to shear force, and the separating force of the fruit stalk was smaller. Thus, when the cutter is used to separate the fruit from the fruit stalk, the optimal angle between the cutter and the fruit stalk ranges from 60° to 90°, which corresponds to a range of 1.3 to 1.7 N of separation cutting force.

### 2.2 Picking requirements and design goals

The design requirements of the kiwifruit picking robot consist of two parts: functional requirements and performance requirements. The functional requirements mainly include: automatically identifying fruit trees, recognizing fruits, gathering fruits, separating fruits from fruit stalks, and collecting fruits. The performance requirements include: automatically adjusting the speed of the mobile device according to the orchard environment, adjusting the angle of the end-effector to recognize fruits, fully aggregating fruits, quickly collecting kiwifruit, and fully adapting to the orchard picking environment.

Currently, the orchard mostly adopts trellis cultivation, which has a larger space underneath and is not impacted by branches, leaves, etc., which is favorable for the implementation of the picking action. According to the physical properties and growth environment of kiwifruit, the working space range of the picking robot is designed as follows: X-direction travel is 1.2~1.5 m, Y-direction travel is 1.5~1.8 m, and Z-direction travel is 1.5~2.0 m. The expected picking indicators are as follows: the fruit picking speed is 7~10 s/cluster, with a fruit picking success rate of more than 90% and a fruit damage rate of less than 10%.

### 2.3 Expected workflow

**Figure 2:**
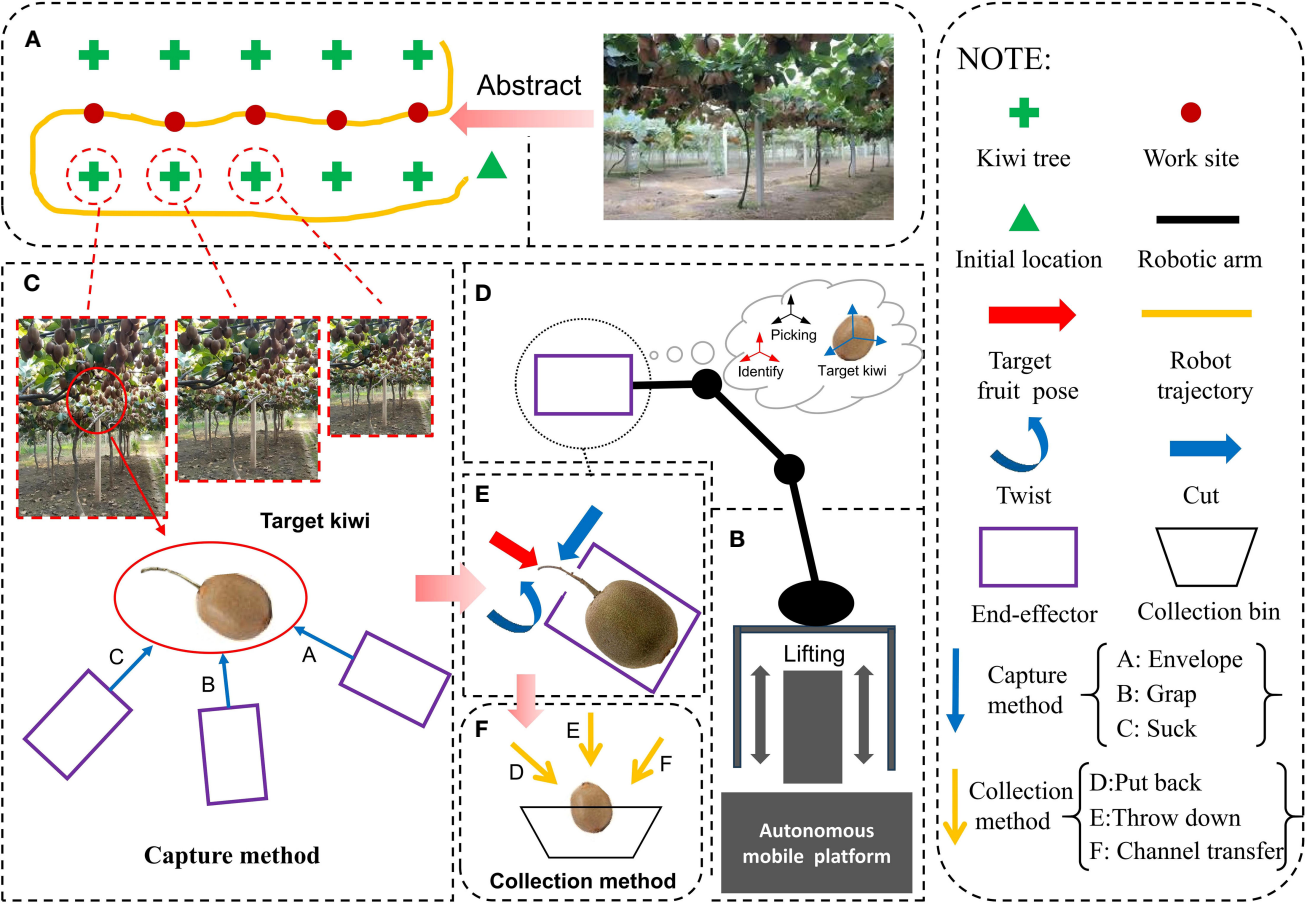
Expected workflow of kiwifruit: **(A)** environmental map of kiwifruit orchards, **(B)** autonomous mobile platforms, **(C)** three methods for end-effector capture of kiwifruits, **(D)** locating the target kiwifruits, **(E)** three methods of separating the fruits from the fruiting stalks, **(F)** three methods of collecting fruits after picking them.

The operation flow of the kiwifruit-picking robot is shown in **Figure 2**. Firstly, the robot enters the kiwifruit orchard via the autonomous mobile platform to locate the target fruit trees. And the end-effector is raised to the suitable kiwifruit-picking area by the lifting device. Subsequently, the end-effector recognizes the fruit by the identification system, approaches the fruit by enveloping or grasping or sucking, and picks the fruit by cutting or pulling or twisting. Finally, the end-effector puts the fruit into a fruit collection box by putting it back or just throwing it down or piping it.

In the operation phase of **Figures 2C–F**, the end-effector fruit-picking process can be divided into three stages: capturing fruits, separating fruit stalks, and collecting fruits. Among them, the methods to capture the fruits mainly include grabbing, sucking, and enveloping; the methods to separate the fruit stalks mainly are cutting, pulling, and twisting; and the key methods to collect the fruits mainly consist of putting them back, throwing them down and collecting them in Pipelines. The specific analysis of the implementation of each stage is shown in **Table 2**.

**Table 2:** End-effector harvesting action method.

	Action	Principle	Characteristic
Capturing fruit method	Envelope	Enveloping of the fruits using a structure with cavities	Multiple fruits can be picked at the same time, suitable for capturing fruits that grow in clusters
	Grab	Simulate human hands to grab the fruits	Picking is highly adaptable and suitable for capturing fruits with harder peels
	Suck	Sucking the fruits to the end-effector by negative pressure	Easy and rapid capturing of fruits, suitable for capturing small shaped fruits
Separating fruit stalks method	Cut	Cutting the fruit stalks using a shearing mechanism	Low damage to fruits and plants, but complicated control system
	Pull	Using external force to pull and break the fruit stalks	Operation is simple, but easy to damage the fruits and plants.
	Twist	Using external force to twist off the fruit stalks	Rapidly twist off fruit stalks, but easily damages fruit skins.
Collecting fruits method	Put back	Using a robotic arm to place fruits into a collection device	Reducing fruit damage but is time consuming
	Throw down	Releasing the fruit directly after picking it from the tree	Easy and convenient to operate, but easy to damage the fruit
	Pipeline	The fruit falls into the collection device through a pipeline	Fruits collection is efficient, but requires the design of piping to match the machine.

In contrast to crowned trees such as apples and citrus, kiwifruit is a vine and is characterized by clustering (see **Figure 1B**). Generally, managers in kiwifruit orchards tend to use a trellis planting pattern, where the fruits hang down on vines and are evenly distributed in the form of clusters.

1. Analyzed for kiwifruit capture methods: If the kiwifruit is captured by grabbing, generally only one fruit can be picked at a time, while the kiwifruit grows in clusters, in order to pick them individually, it is necessary to separate the adjacent fruits. However, the grabbing type end-effector used in this process is prone to damaging the adjacent fruits. If the kiwifruit is captured by suction, two problems may arise. Firstly, if the suction force is too strong, it can easily damage the fruit. On the other hand, if the suction force is too weak, the fruit may easily drop. Secondly, during the suction process, it is likely to suck in the fruit leaves and vines surrounding the fruit into the end-effector. However, when the kiwifruit is captured by means of enveloping, it is possible to address the characteristics of kiwifruit clusters by enveloping the fruit inside the end-effector in the form of fruit clusters, which reduces the rate of damage to the fruit during the fruit capture process.
2. Analyzed for the methods of separating fruit stalk in kiwifruit: Non-destructive and rapid separation of fruit from fruit stalks is the optimal principle for fruit stalk separation. For kiwifruit, this vine plant, the use of pulling for fruit stalk separation can cause damage if the strength is not controlled properly, as it may result in the vine being pulled directly from the trellis and affecting the surrounding fruit. Additionally, since kiwifruit is typically harvested when it is still slightly unripe, the connection between the fruit stalk and the fruit is relatively strong. If the twisted fruit stalk separation method is used, it can easily cause fruit damage. On the other hand, using the cut-off fruit stalk separation method allows for non-destructive separation of the fruit stalk and avoids the occurrence of the aforementioned situations.
3. Analyzed for kiwifruit collection methods:Non-destructive and rapid are also optimal principles for fruit collection. If the robotic arm with the end-effector puts the fruits back into the collection device every time they are picked, it reduces the rate of fruit damage, but it is time-consuming. On the other hand, if the direct throw-down method is used, although it is a rapid collection method, it is easy to damage the fruits. However, with the pipeline collection method, the picked fruits fall directly into the collection device along the conveyor pipeline. This method can compensate for the shortcomings of the above two collection methods and achieve rapid and non-destructive fruit collection.

In summary, after comparative analysis, it was decided in this paper to use enveloping to capture kiwifruits, cutting to separate the fruit stalks, and piping to collect the kiwifruits.

## 3 Structural design

### 3.1 Overall structure

**Figure 3:**
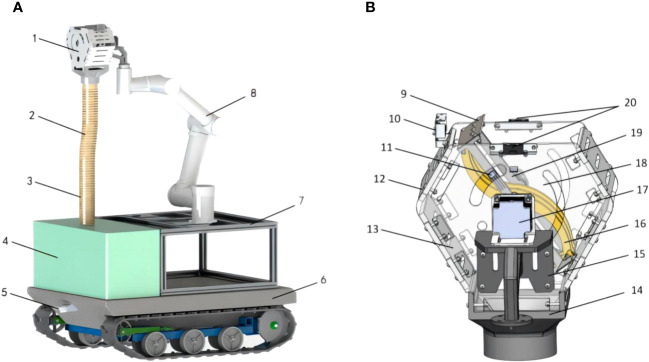
Schematic structure of kiwifruit picking robot: **(A)** overall structure, **(B)** picking end-effector. (1. end-effector 2. retractable hose 3. cushion 4. fruit collection box 5. navigation camera 6. tracked chassis 7. support frame 8. robotic arm 9. cutter 10. photoelectric detection sensor 11. hall sensor 12. baffle plate 13. lateral plate 14. fruit collection end 15. connector 16. curved guiding rod 17. rotary stepper motor 18. picking bin 19. fixing rod 20. infrared recognition sensor).

The overall structure of the kiwifruit picking robot, as shown in **Figure 3A**, is primarily composed of an end-effector, robotic arm, retractable hose, fruits collection box, and crawler chassis. A tracked chassis is used as the mobile device for the kiwifruit-picking robot, allowing it to adapt to the kiwifruit orchard environment and enhancing the robot’s flexibility. A navigation camera is installed at the bottom of the tracked chassis to enable the precise positioning of kiwifruit trees in the orchard. A 6-degree-of-freedom polar coordinate robotic arm has been chosen as a support device for the kiwifruit-picking robot to assist in the end-effector-picking operation. Furthermore, to prevent the kiwifruit’s height during picking from exceeding the robotic arm’s reachable range, a retractable support frame is added beneath the base of the robotic arm to increase the height and optimize space utilization. The collection device utilizes a retractable hose and a fruit collection box. One end of the hose is securely fixed beneath the end-effector, connecting to the envelope-picking bin, while the other end was linked to the fruit collection box.

The end-effector structure, as shown in **Figure 3B**, comprises an external picking bin, an internal rotary cutter mechanism, and a fruit collection end. Two infrared recognition sensors are installed on both sides of the lateral plate to recognize the fruits, and a photoelectric detection sensor is positioned on one side of the baffle plate to determine whether the fruits have entered the picking bin. The rotary cutter mechanism is mainly composed of a rotating shaft, curved guide rods, fixed rods, cutters, and a rotary stepper motor. During operation, the rotary stepper motor drives the cutter mechanism to rotate, causing the cutters to cut off the fruit stalks.

The kiwifruit picking robot parameters is shown in **Table 3**.

**Table 3:** Parameters of the kiwifruit picking robot.

Parameters	Numerical value
Overall dimensions (L×W×H)/m×m×m	2.0×1.5×2.0
Weight of end-effector/kg	4
Retractable length of hose/m	0~0.9
Height of tracked chassis/m	0.6
Retractable length of support frame/m	0~1.0
Lifting height of robotic arm/m	0~0.9
Travel speed of tracked chassis/(m.s^-1^)	0~0.5
Operating speed of robotic arm/(m.s^-1^)	1.5
Speed of stepper motor/(r.min^-1^)	6

### 3.2 Working principle

The working principle of the kiwifruit-picking robot is as follows: The robot initiates system initialization. The robot uses fused SLAM technology based on multiple sensors (including a 16-line 3D LIDAR, IMU and differential GPS/INS navigation module supporting RTK technology) to navigate and localize the fruit trees in the orchard, and uses the Dijkstra’s algorithm and dynamic windowing approach to globally plan the path and avoid dynamic obstacles detected by the LIDAR, and when it reaches the location of the fruit trees, the robot stops moving; The robotic arm delivers the end-effector in the suitable kiwifruit-picking area; Recognition sensors begin scanning the surroundings for kiwifruits, and once they are detected, the robotic arm raises the end-effector from below the kiwifruits to envelope them into the picking bin. When the photoelectric detection sensor confirms that all kiwifruits are inside the picking bin, the robotic arm halts its movement; The rotary cutter turns clockwise to cut off the stalks of the kiwifruits in the picking bin, and kiwifruits fall on curved guide rods; The rotary cutter continues to rotate until it resets, during which time the kiwifruits slowly descend the curved guide rods and drop into the fruit collection end; Finally, the picked kiwifruits are conveyed into the fruit collection box through the retractable hose, completing the single-picking process.

### 3.3 End-effector design

#### 3.3.1 Structural design of picking bin

**Figure 4:**
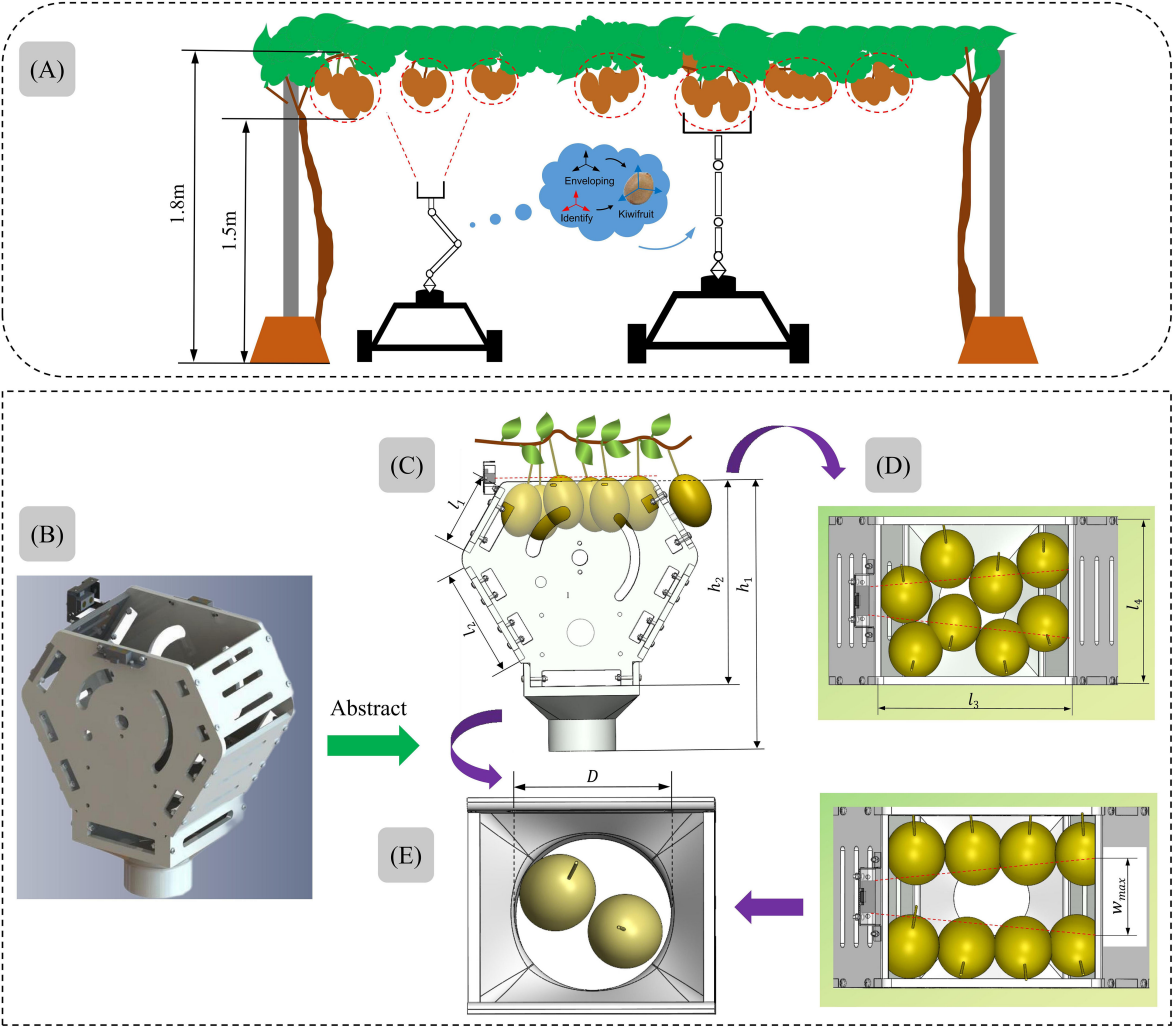
Design of the structural and parameters of the picking bin: **(A)** schematic diagram of picking process, **(B)** structure of the envelope type picking bin, **(C)** the process of enveloping fruits, **(D)** two forms of expected distribution of fruits, **(E)** the fruit collection end transmits the fruits.

The existing kiwifruit picking end-effector mostly leverage a clamping structure to harvest the fruit. However, the clamping structure is susceptible to two issues during the picking process. Firstly, since kiwifruits naturally grow in clusters, the clamping mechanism can only pick one fruit at a time. To achieve single picking, it becomes necessary to separate adjacent fruits first, a process in which the clamping structure may inadvertently damage neighboring fruits. Secondly, when clamping fruits of different sizes, excessive clamping force can lead to fruit damage, while inadequate force can cause the fruits to drop. Aiming at the above problems, and considering that kiwifruit in clusters hangs down on the fruit tree vines in orchards, an envelope-type picking end-effector with clusters as the form of harvesting was proposed in this paper, as shown in **Figure 4A**. During the robot’s picking operation, the end-effector sufficiently envelopes the target fruit clusters from below, and prevents direct contact between the mechanical structure and the fruits. It is expected that by using multi-fruit envelope picking, not only the fruit picking efficiency can be improved, but also the fruit damage rate can be greatly reduced.

The structure of the picking bin is shown in **Figure 4B**, and it consists of two side plates and four baffle plates connected. To prevent damage to fruit outside the picking bin when adjacent fruits are separated, the picking port’s shape has been designed as a “hexagonal shape”, with dimensions $l_{1}$ =90mm, $l_{2}$ =135mm, $h_{1}$ =390mm, $h_{2}$ =270mm, as shown in **Figure 4C**. During the enveloping process, the fruits outside the picking bin are separated from the picked fruits by the baffles. The side plates and baffle plates don’t need to carry heavy loads but require a certain level of toughness to prevent end-effector failure during the picking process. Therefore, 8mm thick carbon fiber plates were chosen for production.

According to the kiwifruit cultivation parameters in section 2.1, kiwifruit has 3~7 fruits per cluster, with the average value of fruit length being 67.2mm, the maximum value being 72.7mm, and the minimum value being 61.4mm; the average value of fruit width being 53mm, the maximum value being 56.7mm, and the minimum value being 49.5mm; the average value of fruit thickness being 47.9mm, the maximum value being 48.3mm, and the minimum value being 43.4mm. In this regard, in order to meet the requirements of multi-fruits picking by the end-effector, as well as to meet the requirements that the fruits in the picking bin can be fully detected by the photoelectric detection sensor (refer to section 3.3.3, the width of detection $w_{max}$ =70mm), the port side of the picking bin was designed to be $l_{3}\times l_{4}$ , i.e. side plate edge length $l_{3}$ =200mm and baffle plate edge length $l_{4}$ =150mm. It is expected that the picking bin port can hold up to about 6 to 8 fruits, as shown in **Figure 4D**. In addition, a fruit collection end was installed below the end-effector to be able to assist in delivering the picked kiwifruits to the fruit collection box. According to the growth parameters of the fruits, the diameter of the fruit collection end was designed to be D= ∅ 120mm, and it is expected that 1~2 fruits can be delivered to the fruit collection box at the same time, as shown in **Figure 4E**.

#### 3.3.2 Parameter design of curved guide rods

**Figure 5:**
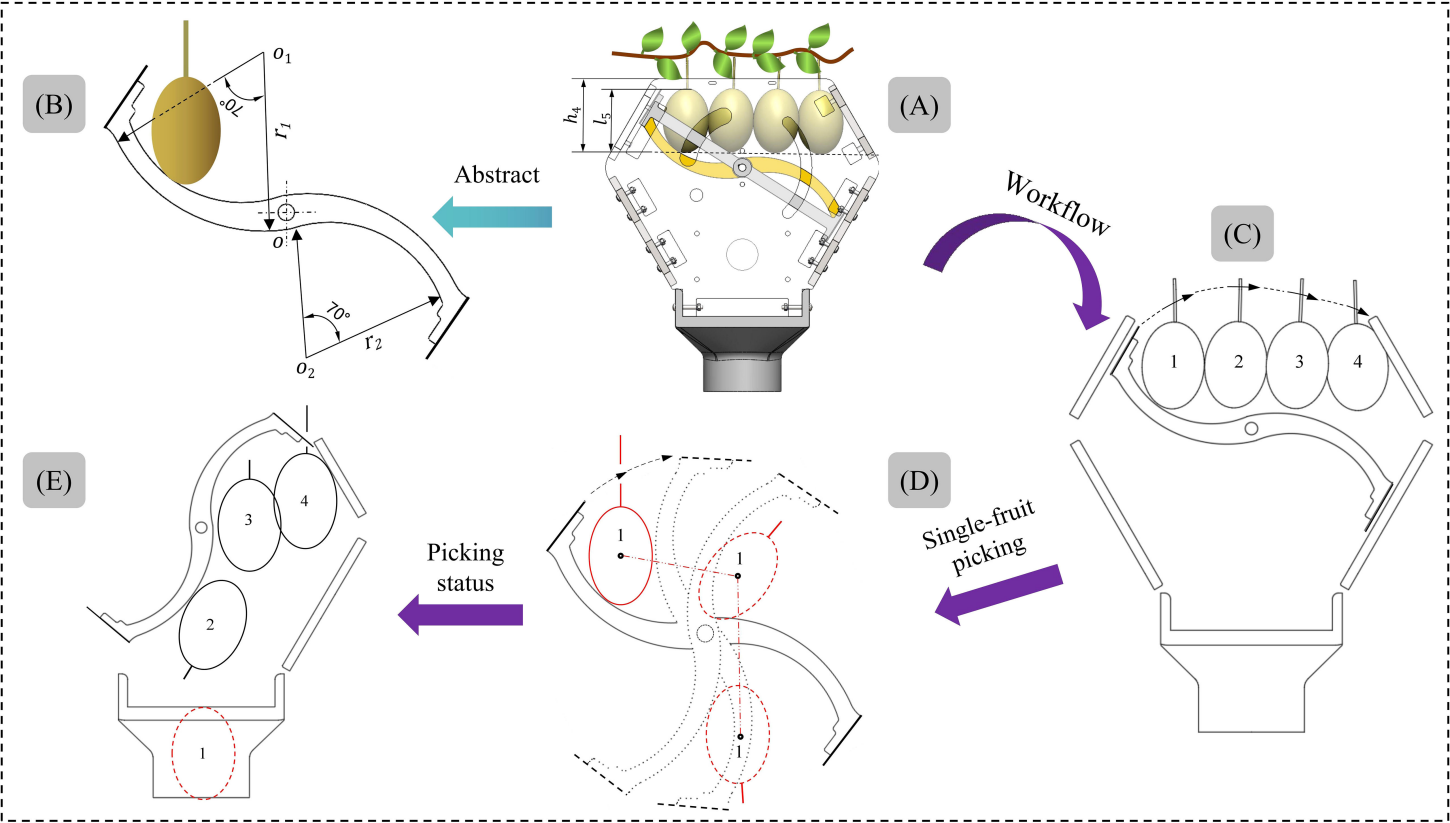
Structure and workflow of curved guide rods: **(A)** limit distribution diagram for side-by-side arrangement of fruits in the picking bin, **(B)** structural diagram of curved guide rod, **(C)** schematic diagram of cutting off all fruit stalks, **(D)** schematic diagram of the curved guide rod delivering fruit 1, **(E)** schematic diagram of the fruits picking state.

The curved guide rod primarily serves as a buffer and takes over the picked fruits during the rotation process, effectively reducing the risk of fruit damage, as shown in **Figure 5B**. Additionally, it plays a guiding role during rotation, assisting the sequentially falling of the picked kiwifruits into the fruit collection end. The curved structure can enhance the smoothness of guidance, and the surface of the curved guide rod is covered with a layer of polyethylene foam cotton, a material known for its softness, excellent toughness, flexibility, and cushioning properties (Zhang et al., 2023). This added layer provides better protection for the fruits, minimizing the risk of damage.

The radius of curvature, width, and other parameters of the curved guide rod have a significant impact on the picking process, given its direct contact with the kiwifruits. If the radius of curvature is too small, there’s a risk of pushing kiwifruits out of the picking bin by the curved guide rod during the enveloping process, potentially causing fruit damage by the cutter. Conversely, if the radius of curvature is too large, it may lead to picked kiwifruits piling up and not passing through the fruit collection end sequentially. The limiting distribution of the side-by-side arrangement of fruits in the picking bin is shown in **Figures 5A, B**, with fruit 1 being the first to touch the curved guide rod when four kiwifruits are at the same height. Therefore, during the enveloping process, as long as it is avoided that fruit 1 is pushed out of the picking bin by the curved guide rod, i.e., the height of the fruit from the port of the picking bin $h_{4}$ is greater than the length of the fruit itself $l_{5}$ , the rest of the fruits will enter the picking bin completely. At this point $h_{4}$ is approximately equal to the curved guide rod inner radius $r_{2}$ . By combining the design parameters of the picking bin and the fruit parameters (refer to section 3.3.1), it was determined that the outer radius of the curved guide rod should be $r_{1}$ =100mm, with an inner radius of $r_{2}$ =72.5mm. The workflow of the curved guide rod is shown in **Figures 5C–E**, where the kiwifruits rotate with the curved guide rod and fall smoothly into the fruit collection end.

#### 3.3.3 Control system design of end-effector

**Figure 6:**
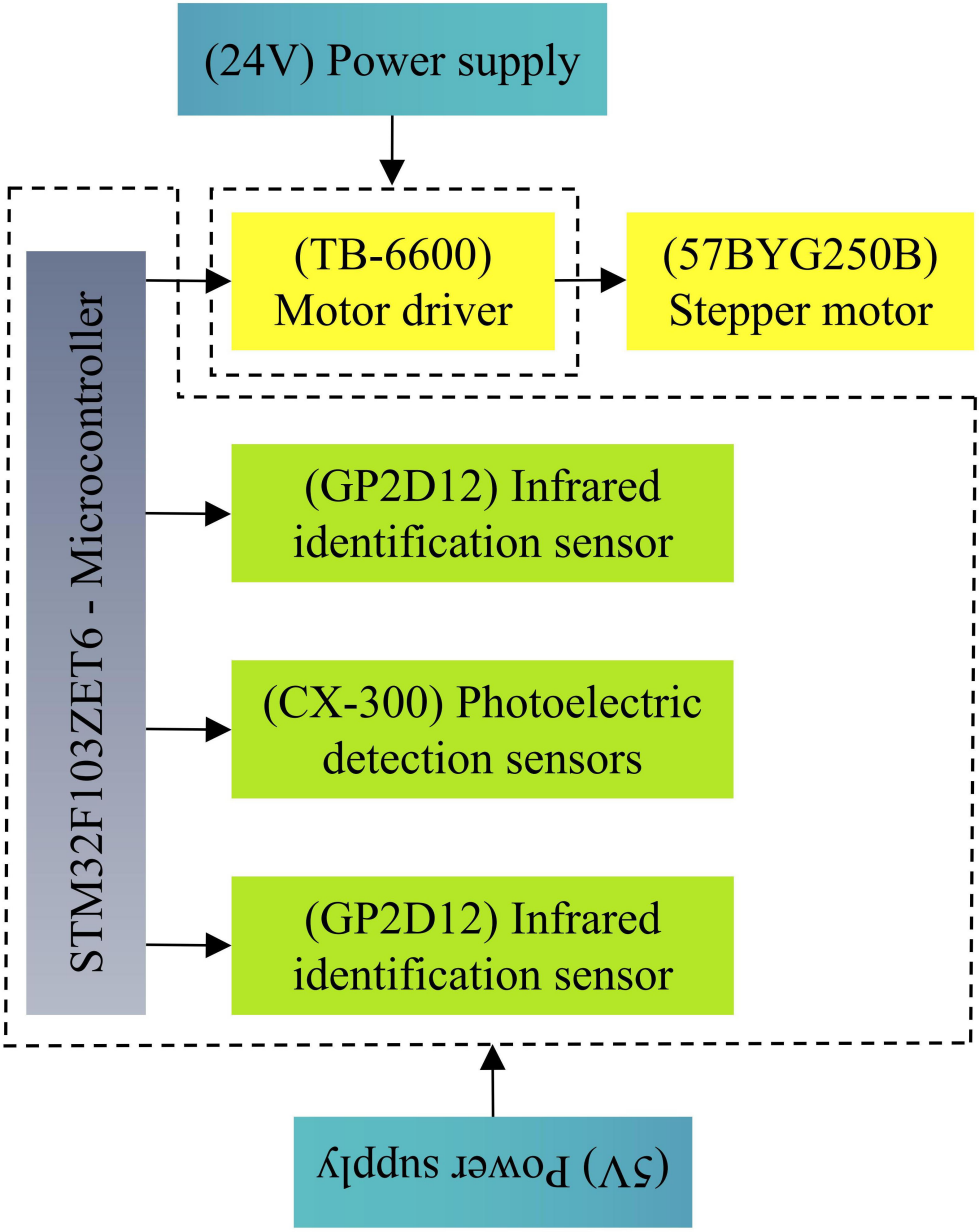
Hardware composition.

The hardware composition of the end-effector control system, as shown in **Figure 6**, includes a controller with an STM32 microcontroller at its core, two infrared recognition sensors, a photoelectric detection sensor, a Hall sensor, a rotary stepper motor, and a motor driver. The control system is powered by two power modules, where the sensor, microcontroller, and motor driver control signal voltage are 5 V, and the stepper motor driver operates at 24 V. The control system was programmed in C language. The microcontroller gathers sensor data, then controls the direction signal, number of pulses, as well as pulse frequency of the stepper motor driver after processing to achieve orderly control of the end-effector actions.

Two infrared identification sensors (Guangdong Xin de Electronics, model GP-2D12) were symmetrically installed on the outer edges of the side plates of the end-effector, as shown in **Figure 3B**. This model of sensor is capable of recognizing fruits within a range of 20 to 80 cm.

The photoelectric detection sensor (Zhongshan Covey Company, model CX-3LIKNP) was installed in the middle of the end-effector single-side baffle, as shown in **Figure 3B**. The signal from this sensor is a bar-shaped colorless light spot, with a detection width range of 10~70mm and a length range of 0~100cm, and only detects preset colors. Typically, matured kiwifruits in the orchard exhibit a brown color, while the fruit stalks are dark green. Therefore, this sensor is set to detect brown. Based on the parameters of the picking bin and fruit parameters (refer to section 3.3.1), the photoelectric detection sensor is set to detect a range of 0 to 20cm. When the kiwifruits enter the picking bin, the sensor triggers an alarm, and the alarm ceases once all the kiwifruits are inside the picking bin. When the alarm stops, the microcontroller commands the stepper motor driver to drive the rotary stepper motor, thereby driving the rotary cutter mechanism to sever the fruit stalks of the fruits in the picking bin.

To prevent the rotary stepping motor from over-traveling, the Hall position sensor (Tianjin Yue er xing Electronic Technology Co., Ltd., YS44E model) was installed on the fixed rod, as shown in **Figure 3B**.

The rotary stepper motor (Guangdong Lv wei Technology Motor Company, model 57BYG250B, motor driver model TB-6600) was installed on the connector and is responsible for driving the rotary cutter mechanism to cut the stalks of the fruits in the picking bin, as shown in **Figure 3B**. This model of stepper motor can provide a thrust of more than 60N and a torque of up to 6N.m at a set speed of 12r/min.

## 4 Kinematic analysis

Robot kinematics is primarily concerned with analyzing the relationship between the position of the end-effector and the joint variables of the robotic arm (Wang K. et al., 2020). This chapter lays the foundation for the trajectory planning and picking strategy of the robot in chapter 5 by calculating the forward and inverse solutions of the robot kinematics and analyzing the workspace, as well as providing theoretical guidance for the motion of the robotic arm during the experiment.

### 4.1 Forward kinematic analysis

**Figure 7:**
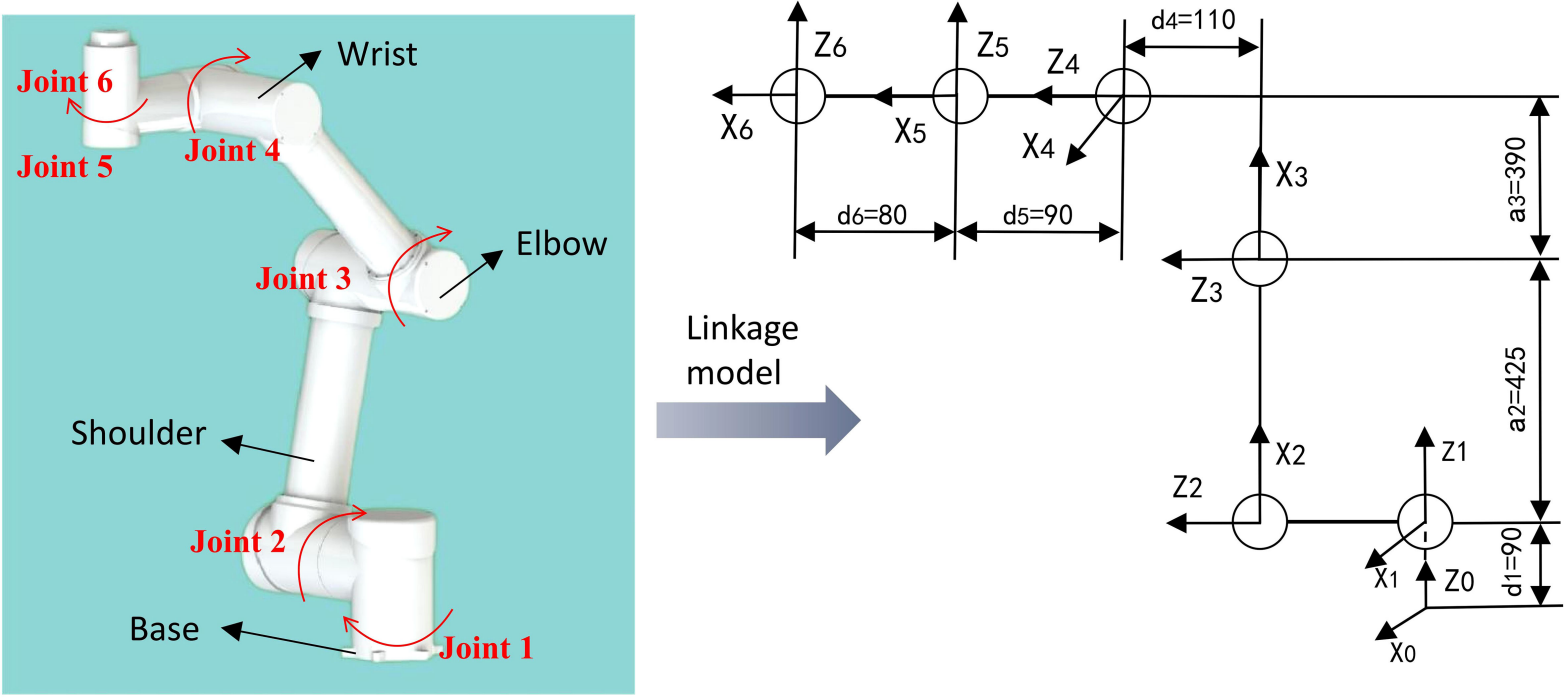
The connecting rod coordinate system.

The kinematic equations of the kiwifruit-picking robot were established using the D-H parameter method, and the connecting rod coordinates are shown in **Figure 7**. The D-H parameters for each joint were determined based on the connecting rod parameters and the relationships within the established coordinate system, as shown in **Table 4**.

**Table 4:** D-H parameters of the robotic arm.

i	${\text{α}}_{\text{i}}$ /(°)	${\text{a}}_{\text{i}}$ /(mm)	$d_{i}$ /(mm)	$\theta_{i}$ /(°)	Variable Range
1	90	0	90	${\text{θ}}_{1}$	-360~360
2	0	425	0	${\text{θ}}_{2}$	-90~90
3	0	390	0	${\text{θ}}_{3}$	-180~180
4	90	0	110	${\text{θ}}_{4}$	-30~30
5	-90	0	90	${\text{θ}}_{5}$	-90~90
6	0	0	80	${\text{θ}}_{6}$	-90~90

*i* is the number of joint, *α_i_*, is the rotation angle of the connecting rod, *α_i_* is the length of the connecting rod, *d_i_* is the offset distance of the connecting rod and θ_i_ is the joint angle. *θ_1_, θ_2_, θ_3_, θ_4_, θ_5_*, and *θ_6_* are joint angle variables.

The transformation matrix Equation 1 and Equation 2 between neighboring connecting rod coordinate systems are:


(1) $${\;}_{\;i}^{i-1}T=\text{Rot}\left(z_{i-1},\theta_{i}\right)\times \text{Trans}\left(z_{i-1},d_{i}\right)\times \text{Trans}\left(x_{i},a_{i}\right)\times \text{Rot}\left(x_{i},\alpha_{i}\right)$$

(2) $${\;}_{\;i}^{i-1}T=\left[\begin{array}{cccc} cos\theta_{i} & -sin\theta_{i}cos\alpha_{i} & sin\theta_{i}sin\alpha_{i} & a_{i}cos\theta_{i}\\ sin\theta_{i} & cos\theta_{i}cos\alpha_{i} & -cos\theta_{i}sin\alpha_{i} & a_{i}sin\theta_{i}\\ 0 & sin\alpha_{i} & cos\alpha_{i} & d_{i}\\ 0 & 0 & 0 & 1 \end{array}\right]$$

Where ${\;}_{\;i}^{i-1}T$ represents the Homogeneous transformation matrix of the connecting rod relative to the connecting rod i−1 ; Trans( ) presents a function of the translation of the coordinate system about the axis; Rot( ) represents a function of the rotation of the coordinate system about the axis. Each connecting rod Substituting the parameters of each connecting rod in **Table 4** into Equation 1 can obtain the transformation matrix Equation 3 of each connecting rod:


(3) $$\begin{array}{c} {\;}_{1}^{0}T=\left[\begin{array}{cccc} c_{1} & 0 & s_{1} & 0\\ s_{1} & 0 & -c_{1} & 0\\ 0 & 1 & 0 & 90\\ 0 & 0 & 0 & 1 \end{array}\right]\;2_{1}^{\;}T=\left[\begin{array}{cccc} c_{2} & -s_{2} & 0 & 425c_{2}\\ s_{2} & c_{2} & 0 & 425s_{2}\\ 0 & 0 & 1 & 90\\ 0 & 0 & 0 & 1 \end{array}\right]\;3_{2}^{\;}T=\left[\begin{array}{cccc} c_{3} & -s_{3} & 0 & 390c_{3}\\ s_{3} & c_{3} & 0 & 390s_{3}\\ 0 & 0 & 1 & 0\\ 0 & 0 & 0 & 1 \end{array}\right]\\ 4_{3}^{\;}T=\left[\begin{array}{cccc} c_{4} & 0 & s_{4} & 0\\ s_{4} & 0 & -c_{4} & 0\\ 0 & 1 & 0 & 110\\ 0 & 0 & 0 & 1 \end{array}\right]\;5_{4}^{\;}T=\left[\begin{array}{cccc} c_{5} & 0 & -s_{5} & 0\\ s_{5} & 0 & c_{5} & 0\\ 0 & -1 & 0 & 90\\ 0 & 0 & 0 & 1 \end{array}\right]\;{\;}_{6}^{5}T=\left[\begin{array}{cccc} c_{6} & -s_{6} & 0 & 0\\ s_{6} & c_{6} & 0 & 0\\ 0 & 0 & 1 & 80\\ 0 & 0 & 0 & 1 \end{array}\right] \end{array}$$

Where the kiwifruit picking robot equation of motion T60 is expressed as the position of the end-effector with respect to the base coordinate system:


(4)  60T=T10T21T32T43T54T65=[nxoxaxpxnyoyaypynzozazpz0001]

Where $c_{i}$ is an abbreviation of $cos\theta_{i}$ , $s_{i}$ is an abbreviation of $sin\theta_{i}$ , T represents the corresponding change matrix, T10 , T21 , T32 , T43 , T54 , represent the change matrices for each joint, respectively. T50 is represented as the position of the end-effector relative to the base coordinate system.

To simplify writing, letting: $cos\;(\theta_{I\;}+\theta_{j})$ *=* $c_{ij}$ , $sin\;(\theta_{I\;}+\theta_{j})$ = $s_{ij}$ , $cos\;(\theta_{I\;}+\theta_{j}+\theta_{k})$ = $c_{ijk}$ , $cos\;(\theta_{I\;}+\theta_{j}+\theta_{k})$ = $c_{ijk}$

And by substituting them into Equation 4, Equation 5 can be obtained:

Among them:


(5) $$\left\{ \begin{array}{l} n_{x}=c_{6}\left(s_{1}s_{5}+c_{5}c_{1}c_{234}\right)-s_{6}c_{1}s_{234}\\ n_{y}=c_{6}\left(c_{5}s_{1}c_{234}-c_{1}s_{5}\right)-s_{6}s_{5}s_{234}\\ n_{z}=c_{5}c_{6}s_{234}+s_{6}c_{234}\\ o_{x}=-s_{6}\left(c_{5}c_{1}c_{234}+s_{1}s_{5}\right)-c_{6}c_{1}s_{234}\\ o_{y}=-s_{6}\left(c_{5}s_{1}c_{234}-c_{1}s_{5}\right)-c_{6}s_{1}s_{234}\\ o_{z}=c_{6}c_{234}-c_{5}s_{6}s_{234}\\ a_{x}=-s_{5}c_{1}c_{234}+c_{5}s_{1}\\ a_{y}=-s_{5}s_{1}c_{234}-c_{1}c_{5}\\ a_{z}=-s_{5}s_{234}\\ p_{x}=90c_{1}s_{234}+110s_{1}+80\left(c_{5}s_{1}-s_{5}c_{1}c_{234}\right)+425c_{1}c_{2}+390c_{1}c_{23}\\ p_{y}=90s_{1}s_{234}-110c_{1}-80\left(s_{5}s_{1}c_{234}+c_{1}c_{5}\right)+425c_{2}s_{1}+390s_{1}c_{23}\\ p_{z}=90-90c_{234}+425s_{2}+390s_{23}-80s_{5}s_{234} \end{array}\right.$$

### 4.2 Inverse kinematic analysis

The kinematic inverse solution is that the angle of each joint is calculated by knowing the position and attitude of the robot’s end-effector to achieve precise positioning and control of the robot. In this section, the matrix inverse multiplication analytic method was used to solve the kinematic inverse solution of the kiwifruit picking robot.

(1) Solving for $\theta_{1}$ 、 $\theta_{5}$ and $\theta_{6}$ :

Multiply both sides of Equation 4 to the left by T10 −1T65 −1 :


(6) T10 -1T60T65 -1=T21T32T43T54

The left side of the equation:


$$\left[\begin{array}{cccc} c_{6}\left(n_{x}c_{1}+n_{y}s_{1}\right)-s_{6}\left(o_{x}c_{1}+o_{y}s_{1}\right) & s_{6}\left(n_{x}c_{1}+n_{y}s_{1}\right)+c_{6}\left(o_{x}c_{1}+o_{y}s_{1}\right) & a_{x}c_{1}+a_{y}s_{1} & p_{x}c_{1}-80\left(a_{x}c_{1}+a_{y}s_{1}\right)+p_{y}s_{1}\\ n_{x}c_{6}-o_{z}s_{6} & o_{z}c_{6}+n_{z}s_{6} & a_{z} & p_{z}-90-80a_{z}\\ s_{6}\left(o_{y}c_{1}-o_{x}s_{1}\right)-s_{6}\left(n_{y}c_{1}-n_{x}s_{1}\right) & -s_{6}\left(n_{y}c_{1}-n_{x}s_{1}\right)-c_{6}\left(o_{y}c_{1}-o_{x}s_{1}\right) & a_{x}s_{1}-a_{y}c_{1} & -p_{y}c_{1}+80\left(a_{y}c_{1}-a_{x}s_{1}\right)+p_{x}s_{1}\\ 0 & 0 & 0 & 1 \end{array}\right]$$

The right side of the equation:


$$\left[\begin{array}{cccc} c_{234}c_{5} & -s_{234} & -c_{234}s_{5} & a_{3}c_{23}+a_{2}c_{2}+90s_{234}\\ s_{234}c_{5} & c_{234} & -s_{234}s_{5} & a_{3}s_{23}+a_{2}s_{2}-90c_{234}\\ s_{5} & 0 & c_{5} & 110\\ 0 & 0 & 0 & 1 \end{array}\right]$$

According to the equality of the corresponding row 3, column 4 on the left and right sides of Equation 6, it can be seen Equation 7:


(7) $$-p_{y}c_{1}+80\left(a_{y}c_{1}-a_{x}s_{1}\right)+p_{x}s_{1}=110$$

Organize and obtain Equation 8:


$$\left(80a_{y}-p_{y}\right)c_{1}-\left(80a_{x}-p_{x}\right)s_{1}=110,\text{ and letting }80a_{y}-p_{y}=m,\;80a_{x}-p_{x}=n$$

(8) $$\theta_{1}=A\tan2(m,n)-A\tan2(110,\pm \sqrt{m^{2}+n^{2}-110^{2}})$$

From Equation 6, Equation 9 can be obtained $\left\{ \begin{array}{l} a_{x}s_{1}-a_{y}c_{1}=c_{5}\\ s_{6}\left(o_{y}c_{1}-o_{x}s_{1}\right)-c6\left(n_{y}c_{1}-n_{x}s_{1}\right)=s_{5} \end{array}\right.$ :


(9) $$\left\{ \begin{array}{l} \theta_{5}=\pm \arccos\left(a_{x}s_{1}-a_{y}c_{1}\right)\\ \theta_{6}=A\tan2(\frac{n_{x}s_{1}-n_{y}o_{x}}{s_{5}},\frac{o_{x}s_{1}-o_{y}c_{1}}{s_{5}}) \end{array}\right.$$

(2) Solving for $\theta_{2}$ 、 $\theta_{3}$ and $\theta_{4}$ :

Multiply both sides of Equation 4 to the right by T54 −1 :


(10) T10 -1T60T65 -1T54 -1= 21T32 T43 T

Using Equation 10 the left and right sides of row 1, column 4 correspond to be equal, and row 2, column 4 correspond to be equal can be shown:


(11) $$\left\{ \begin{array}{l} 90\left(s_{6}\left(n_{x}c_{1}+n_{y}s_{1}\right)+c_{6}\left(o_{x}c_{1}+o_{y}s_{1}\right)\right)-90\left(a_{x}c_{1}+a_{y}s_{1}\right)+p_{x}c_{1}+p_{y}s_{1}=a_{3}c_{23}+a_{2}c_{2}\\ p_{z}-90-80a_{z}+90\left(o_{z}c_{6}+n_{z}s_{6}\right)=a_{3}s_{23}+a_{2}s_{2} \end{array}\right.$$

Combined with the above method of finding the value, according to Equation 11 can be obtained Equation 12:


(12) $$\left\{ \begin{array}{l} \theta_{3}=\pm \arccos\left(\frac{m_{2}+n_{2}-a_{2}{\;}^{2}-a_{3}{\;}^{2}}{2a_{2}a_{3}}\right)\\ \theta_{2}=A\tan2\left(\frac{\left(a_{3}c_{3}+a_{2}\right)n-a_{3}s_{3}m}{a_{2}{\;}^{2}+a_{3}{\;}^{2}+2a_{2}a_{3}c_{3}},\frac{m+a_{3}s_{3}s_{2}}{a_{3}c_{3}+a_{2}}\right)\\ m=90\left(s_{6}\left(n_{x}c_{1}+n_{y}s_{1}\right)+c_{6}\left(o_{x}c_{1}+o_{y}s_{1}\right)\right)-90\left(a_{x}c_{1}+a_{y}s_{1}\right)+p_{x}c_{1}+p_{y}s_{1}\\ n=p_{z}-90-80a_{z}+90\left(o_{z}c_{6}+n_{z}s_{6}\right) \end{array}\right.$$

According to the equality of the corresponding 2nd row, 2nd column and 1st row, 2nd column on the left and right sides of Equation 6, it can be seen Equation 13: 


(13) $$\theta_{4}=A\tan2\left(-s_{6}\left(n_{x}c_{1}+n_{y}s_{1}\right)-c_{6}\left(o_{x}c_{1}+o_{y}s_{1}\right),o_{z}c_{6}+n_{z}s_{6}\right)-\theta_{2}-\theta_{3}$$

### 4.3 Workspace analysis

**Figure 8:**
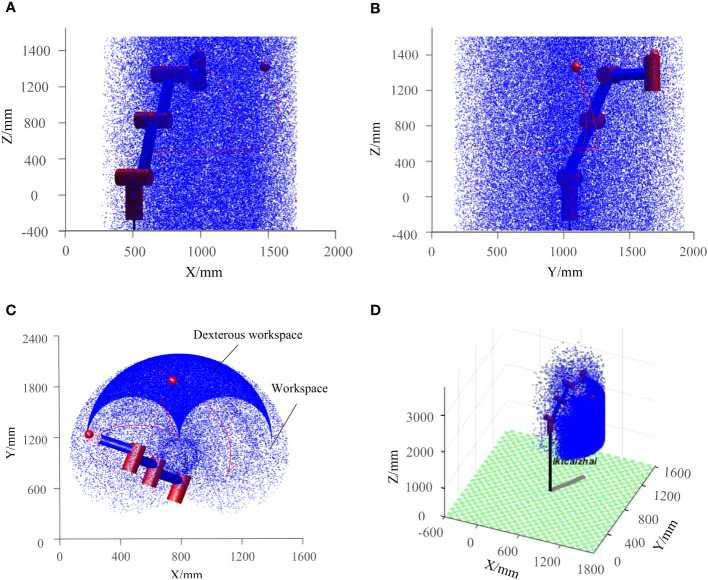
Workspace of the kiwifruit picking robot: **(A)** XOZ planar projection, **(B)** YOZ planar projection, **(C)** XOY planar projection, **(D)** Three-dimensional space.

The workspace is the collection of spatial points that can be reached by the end-effector during the picking process of the robot, and its shape and size are the key factors that determine the robot’s work performance. Based on the Robotics Toolbox in MATLAB software, the Monte Carlo method was used to solve the approximate solution in motion space (Xu Z. et al., 2018). Through the forward kinematics Equation 4, the position vector of the end-effector relative to the base was solved, and then multiple end position coordinate points were randomly generated by the Rand function to obtain the workspace as shown in **Figure 8**.

From **Figure 8**, the workspace of the kiwifruit-picking robot is a heart-like columnar space, X-direction travel is about 1.4 m; Y-direction travel is about 1.8 m; and Z-direction travel is about 2.0 m. And the dexterous workspace is shown in **Figure 8C**. The space avoids collisions between the robotic arm and the internal branches of the kiwifruit tree, as well as interference between the arm and the mechanics of the robot. During the actual picking task, the position of the picked kiwifruit should be within the dexterity workspace. The simulation results provide the foundation for the trajectory planning of the robotic arm and the design of the picking strategy in the subsequent section.

## 5 Picking trajectory planning

When the robot has finished picking kiwifruit i−1 and will pick kiwifruit i , the picking motion can be divided into three phases:

1. Leaving the kiwi i−1 place, avoiding branches and leaves.
2. Moving within a safe area where there will be no collision with the branching vine, approaching the target kiwi i .
3. Arriving at the target kiwi, the end-effector performs the picking.

**Figure 9:**
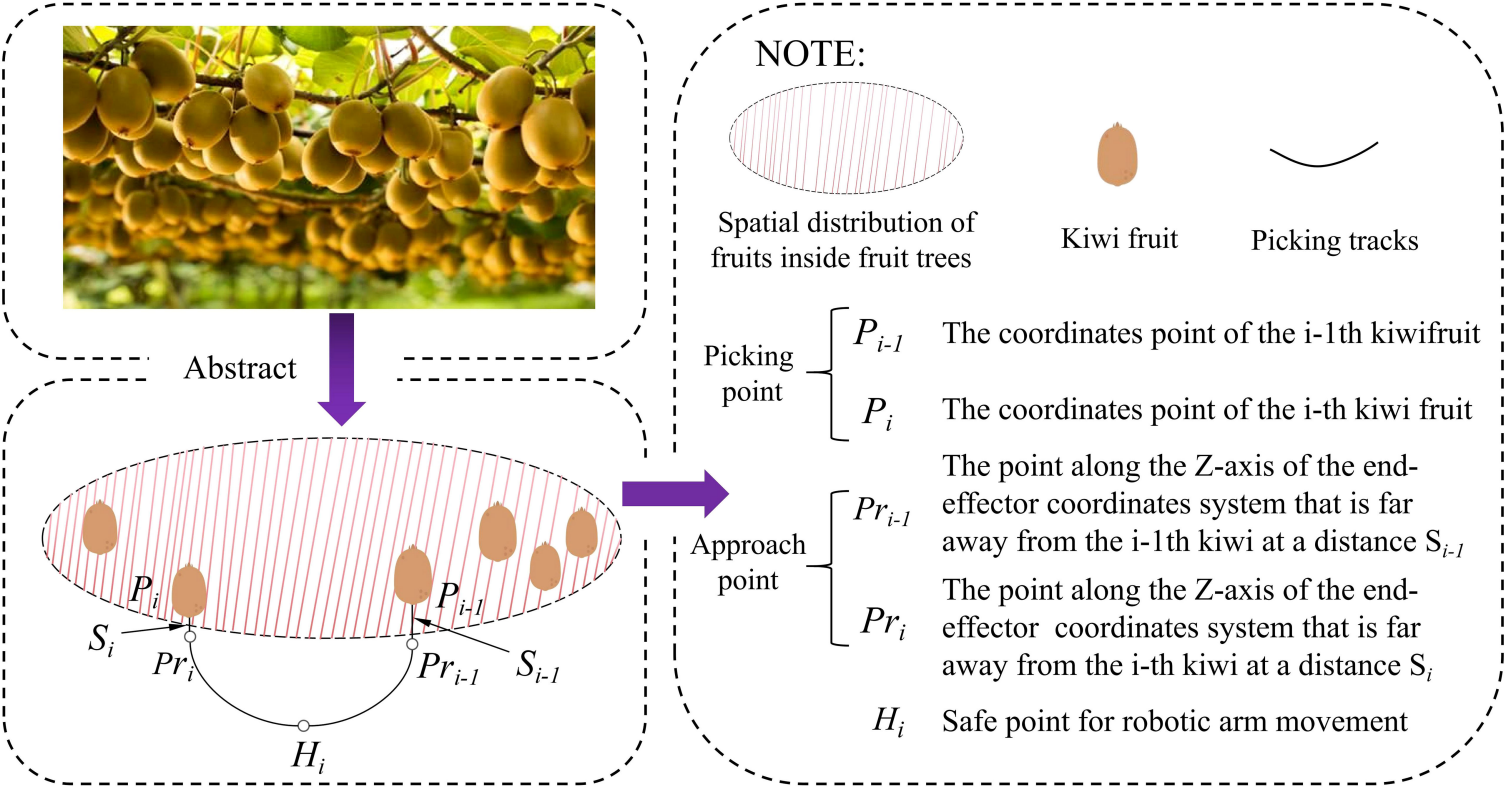
Trajectory planning node between two picks.

During the transfer of the picking target, it was necessary to ensure that the branches of the fruit tree don’t interfere with the robot. During trajectory planning, a safety point H was added to split the motion of the second stage into two parts to ensure that the positional transitions of the robotic arm could take place in a safe area. As a result, each picking task involved the planning of four trajectory segments and five points, as illustrated in **Figure 9**.

When i≠0 , the robot has just finished picking kiwifruit i−1 and will pick kiwifruit  i . The path for trajectory planning is:


$$P_{i-1}\to Pr_{i-1}\to H_{i}\to Pr_{i}\to P_{i}$$

When i=0 , the robot has just started to work, the robot arm is in the zero position, and the coordinates position of the end-effector is the point H at this time. The path for trajectory planning is:


$$H_{0}\to Pr_{0}\to P_{0}$$

### 5.1 Trajectory planning of the robotic arm

The purpose of robotic arm trajectory planning is to ensure that the position, velocity, and acceleration function curves of each joint variable are continuous and smooth during the completion of operational tasks (Yang and Cheng, 2018). When the kiwifruit-picking robot carries out fruit-picking work, the movement of the end-effector should follow to a specific trajectory. The continuous trajectory function minimizes the impact vibrations on each joint of the robotic arm during operation, resulting in smoother robot operation. To meet actual requirements, joint space trajectory planning was implemented for real-time control of the robotic arm by adjusting each joint angle.

Knowing the joint position, velocity, and acceleration at the initial and termination moments, a fifth-degree polynomial can be used to ensure smooth continuity of the joint angle, angular velocity, and angular acceleration, that is:


(14) $$\theta (t)=a_{0}+a_{1}t+a_{2}t^{2}+a_{3}t^{3}+a_{4}t^{4}+a_{5}t^{5}$$

If the six constraints on the angle, angular velocity, and angular acceleration of the initial and terminating joints are:


(15) $$\theta \left(t_{0}\right)=\theta_{0},\theta \left(t_{f}\right)=\theta_{f},\dot{\theta }\left(t_{0}\right)={\dot{\theta }}_{0},\dot{\theta }\left(t_{f}\right)={\dot{\theta }}_{f},\ddot{\theta }\left(t_{0}\right)={\ddot{\theta }}_{0},\ddot{\theta }\left(t_{f}\right)={\ddot{\theta }}_{f}$$

By solving for the first and second-order derivatives of Equation 14, the angular velocity function can be obtained as:


(16) $$\left\{ \begin{array}{l} \theta_{f}=a_{0}+a_{1}t+a_{2}t^{2}+a_{3}t^{3}+a_{4}t^{4}+a_{5}t^{5}\\ {\dot{\theta }}_{f}=a_{1}+2a_{2}t+3a_{3}t^{2}+4a_{4}t^{3}+5a_{5}t^{4}\\ {\ddot{\theta }}_{f}=2a_{2}+6a_{3}t+12a_{4}t^{2}+20a_{5}t^{3} \end{array}\right.$$

Combining Equations 14–16, the coefficients of the fifth-degree polynomial can be obtained as Equation 17:


(17) $$\left\{ \begin{array}{l} a_{0}=\theta_{0}\\ a_{1}={\dot{\theta }}_{0}\\ a_{2}=\frac{{\ddot{\theta }}_{0}}{2}\\ a_{3}=\frac{20\theta_{f}-20\theta_{0}-(8{\dot{\theta }}_{f}+12{\dot{\theta }}_{0})t_{e}-(3{\ddot{\theta }}_{0}-{\ddot{\theta }}_{f})t_{f}^{2}}{2t_{f}^{3}}\\ a_{4}=\frac{30\theta_{0}-30\theta_{f}+(14{\dot{\theta }}_{f}+16{\dot{\theta }}_{0})t_{e}-(3{\ddot{\theta }}_{0}-2{\ddot{\theta }}_{f})t_{f}^{2}}{2t_{f}^{4}}\\ a_{5}=\frac{12\theta_{f}-12\theta_{0}-(6{\dot{\theta }}_{f}+6{\dot{\theta }}_{0})t_{e}-({\ddot{\theta }}_{0}-{\ddot{\theta }}_{f})t_{f}^{2}}{2t_{f}^{5}} \end{array}\right.$$

A time-varying function is utilized to represent each joint variable during joint space trajectory planning, by which the process of trajectory change of the robot over a certain period of time is illustrated. The start point coordinate ${\text{q}}_{0}$ and end point coordinate ${\text{q}}_{1}$ of the robot are given.Utilizing thefunction, the call format is Equation 18:


(18) $$\left[\begin{array}{ccc} \dot{o} & \dot{p} & \dot{q} \end{array}\;\right]=jtraj\left({\text{q}}_{0},{\text{q}}_{1},\text{t}\right)$$

**Figure 10:**
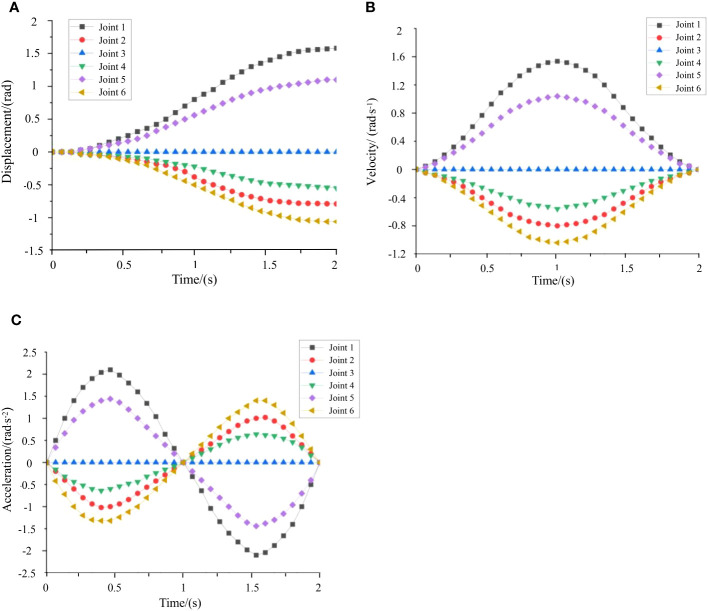
Dynamics of the joints over time: **(A)** displacement curve, **(B)** velocity curve, **(C)** acceleration curve.

Where: $\dot{o}$ stands for displacement; $\dot{p}$ stands for velocity; $\dot{q}$ stands for acceleration; t stands for simulation time. The function jtraj is a fifth-degree polynomial interpolation, with default initial and final velocities set to zero. The simulation time was 2s, and the jtraj function was called to observe the motion change process of each joint. The plot function was then used to generate the displacement, angular velocity, and angular acceleration curves of the robot, as shown in **Figure 10**.

**Figure 10A** reflects the movement process of each joint over time. **Figure 10B** reflects the velocity variation of each joint over time, and they satisfy the requirement that the starting and ending velocities of the fifth-degree polynomial interpolation are all zero. The change in acceleration for each joint is depicted in **Figure 10C** and the curved motion is smooth without abrupt changes. Velocity and acceleration curves are smooth and excessive, without inflection points, interruptions, jumps, and other phenomena, indicating that the robot ran smoothly.

### 5.2 Calculation of critical nodes of the path

There are several critical nodes in the path of picking: picking point $P_{i-1}$ , picking point $P_{i}$ , approach point $\;Pr_{i-1}$ , approach point $Pr_{i}$ , safety point $H_{i}$ . The critical nodes constrain the position in the picking path to avoid the interference of branches and leaves to the robotic arm. Therefore, the position of critical nodes directly affects the motion trajectory of the robot.

The critical nodes can be designed by the RRT (Rapidly Expanding Randomized Tree) algorithm. RRT algorithm is a random sampling algorithm that uses the starting point as the root node, increases the number of nodes by random same, piling, and connects the nodes to generate a random tree (Li et al., 2020; Wang J. et al., 2020; Wang L. et al., 2023). The nodes that do not satisfy the constraint requirements are discarded during the generation of the next node. When the random tree is able to contain the goal point or enter within the goal area, then there exists a route that connects the start point to the endpoint.

The critical nodes in the picking path were computed using the RRT algorithm.Settling the current picking position as $p_{i}$ , random sampling in the free space to get the node as $p_{rand1}$ , at this time there are only two nodes $p_{i}$ and $p_{rand1}$ , so $p_{i}$ that is the closest node to $p_{rand1}$ . Connecting the two nodes and getting the set *m* (k is a coefficient) of the connecting line transits, which is Equation 19:


(19) $$m=\left\{m|m=p_{i}+k\left(p_{rand}-p_{i}\right)\right\}$$

If the path point *m* crosses the obstacle space $C_{0}$ , which is Equation 20:


(20) $$m\in C_{0}$$

Then the node $p_{rand1}$ is discarded and a new node $p_{rand2}$ is generated from scratch. If (as shown in Equation 21)


(21) $$m\notin C_{0}$$

Then grow one step from $p_{i}$ towards $p_{rand}$ to get the new node $p_{i+1}$ . Then continuing to randomly generate new nodes, at this time it is necessary to determine the nearest node with which to connect, so the distance *d* is calculated as Equation 22:


(22) $$d=\|p_{rand3}-p_{k}\|$$

After computational comparison, $p_{rand3}$ selected the closest node for connection and followed the same method as before to determine the obstacle space of the path and finally generate a new node. By analogy, all path nodes before reaching the next picking point can be obtained.

**Figure 11:**
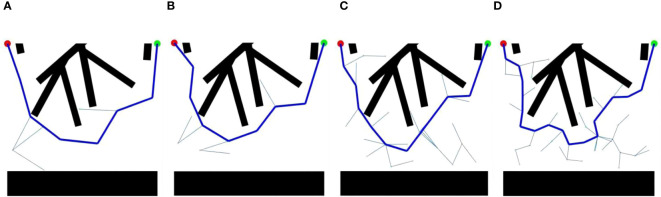
Generation of critical nodes in the picking path: **(A)** step 100, **(B)** step 80, **(C)** step 60, **(D)** step 40.

The environmental parameters during the kiwifruit picking cycle were set and the critical nodes were calculated using the RRT algorithm program. The simulation results are shown in **Figure 11**, which shows that the upper black area simulates the branch obstacle space, and the lower black area simulates the body of the kiwifruit-picking robot, with the red dots in the upper left corner representing the last kiwifruit picked, and the green dots in the upper right corner representing the next kiwifruit to be picked.

### 5.3 Dynamic simulation of picking trajectory

**Figure 12:**
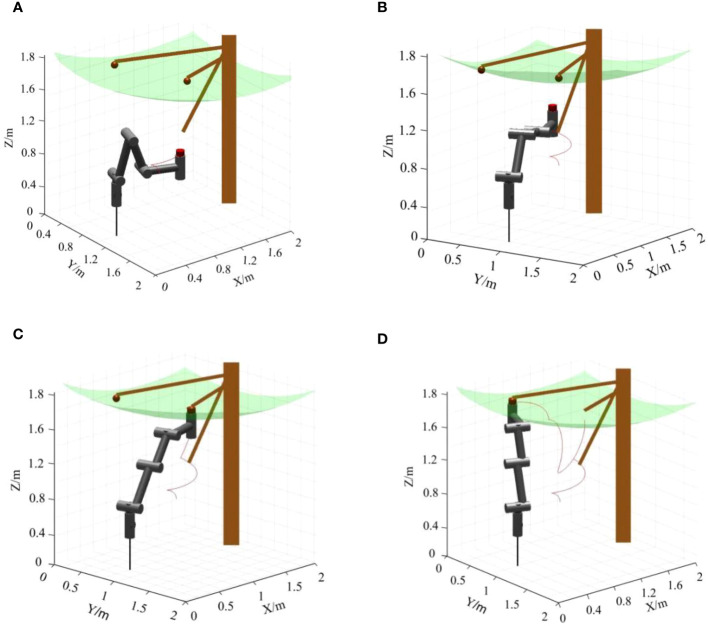
Dynamic simulation of the picking trajectory: **(A)** t=0 s, **(B)** t=4 s, **(C)** t=8 s, **(D)** t=16 s.

Based on the kinematic forward and inverse solutions and the workspace analysis of the picking robot in Chapter 4, the MATLAB software was applied to simulate the trajectory of the kiwifruit picking robot. Two clusters of fruits located at different heights on a kiwifruit tree were used as an example to simulate the growth of kiwifruit grown on a trellis in an orchard, and the simulation results are shown in **Figure 12** (Brown areas represent kiwifruit trees, red areas represent kiwifruit clusters, and green areas are modeled as the spatial distribution of fruit).

Starting from recognizing the first cluster of kiwifruits and ending with recognizing the second cluster of kiwifruits, the robot took a total time of 16 seconds. At t = 0 s, the robot is in the initial state. At 1~4 s, the robot follows the preset picking route, and the robotic arm smoothly avoids the branches and leaves and delivers the end-effector to the fruit picking area. At 5-8 s, the end-effector recognizes the first cluster of fruits and completes the harvesting of the first fruit cluster by enveloping the fruits, cutting off the fruit stalks, collecting the fruits, and other actions. At 9-16 s, the robotic arm first returns to the safe area and then follows the preset picking route to recognize the second cluster of kiwifruits and complete the picking without disturbing other fruits. The simulation results show that the time for the robot to pick a cluster of fruits was 8s, and the picking trajectory met the requirements of the kiwifruit picking operation space.

## 6 Test and results

### 6.1 Test bed set Up

**Figure 13:**
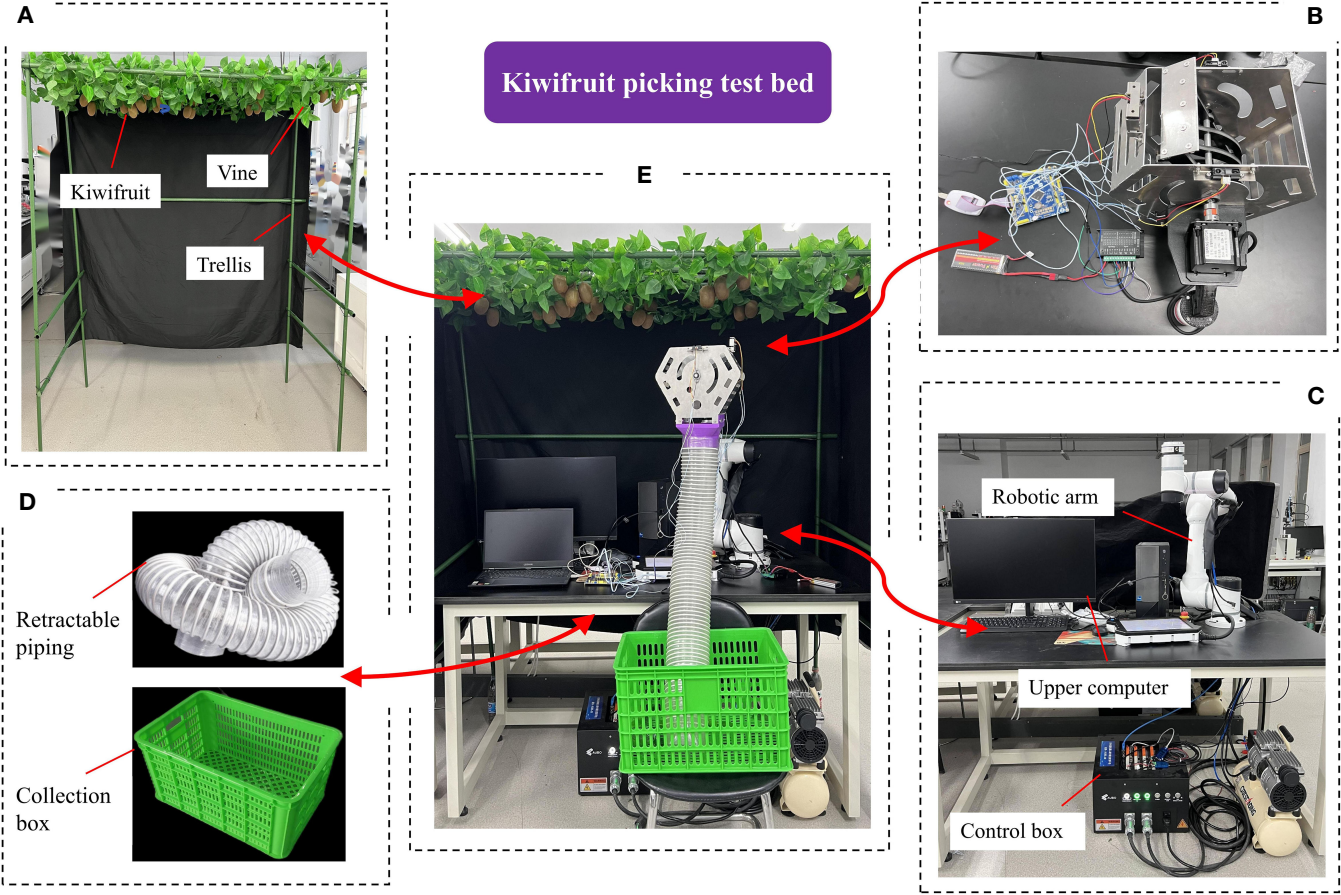
Kiwifruit harvesting test bed: **(A)** trellis model, **(B)** end-effector, **(C)** AUBO-E5 test bed, **(D)** collection device, **(E)** test scenarios.

In the later experimental phase of this study, due to the influence of kiwifruit fruit harvesting season, a test bed was set up in the laboratory environment to simulate a kiwifruit orchard for the picking experiment, as shown in **Figure 13**. The test bed mainly includes: the AUBO-E5 robotic arm test bed, trellis model, the kiwifruit picking end-effector and its control system, Leno R7000P portable computer, collection device, etc.

### 6.2 Test method

The picking experiment was carried out on August 3, 2023. AUBO-E5 robotic arm was used to assist the end-effector operation, and the picking operation flow is shown in **Figure 7** (refer to section 3.2). In this paper, 125 kiwifruits of different shapes were selected for testing to verify the harvesting performance of the end-effector. Based on the characteristics of kiwifruit clusters, 125 fruits were divided into 5 groups according to the number of fruits per cluster: 3, 4, 5, 6, and 7, and each group had 5 clusters with the same number of fruits. In this section, the picking success rate, average picking time per picking, and picking damage rate were used as the evaluation indexes for fast and non-destructive picking. The definitions are shown in Equations 23–25 below:


(23) $$T=\frac{t_{0}-t_{1}}{5}$$

(24) $$S=\frac{n}{h}\times 100\%$$

(25) $$D=\frac{m}{n}\times 100\%$$

Where *T* is the average picking time (includes recognizing fruit clusters, enveloping fruit clusters, and cutting the fruit stalks of the fruit in the picking bin); *t_0_* is the time of the start of picking; *t_1_* is the time of the end of picking; *S* is the success rate; *n* is the number of fruits picked successfully; *h* is the total number of fruits; *D* is the damage rate of fruits picked successfully; and *m* is the number of damaged fruits.

### 6.3 Analysis of results

**Figure 14:**
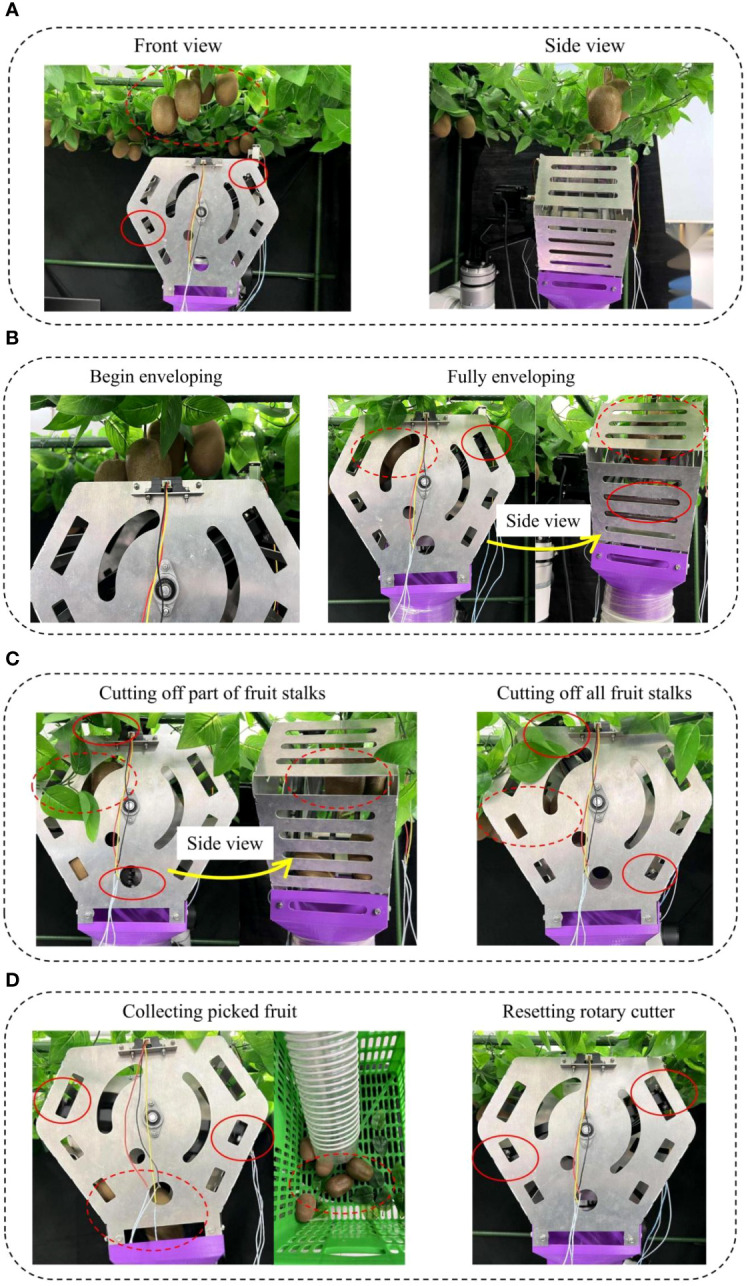
Kiwifruit picking process: **(A)** recognizing the fruits (front view and side view), **(B)** the process from the beginning of the envelopment of the fruit to the complete envelopment of the fruit, **(C)** the process of rotating the cutter mechanism to cut off all fruit stalks in the picking bin, **(D)** during the resetting process, the picked kiwifruits fall through the fruit collection end along the hose into the fruit collection box. (represents rotary cutter real-time status, represents fruit real-time status).

Picking experiments were carried out on 5 groups of 25 clusters of fruits respectively, as shown in **Figure 14**. The robotic arm first sent the end-effector to the area suitable for picking fruits, and after the fruits were detected by the recognition sensor, the robotic arm carried the end-effector upwardly to envelop and pick the fruits, and the picked fruits fell into a fruit collection box through the hose.

During the above fruit-picking process, the robotic arm carried the end-effector picking operation at a speed of 1.5 m/s, and the stepper motor carried the rotary cutter at a speed of 6 r/min to separate the fruits from the fruit stalks, and the results of the picking experiments as shown in **Table 5**.

**Table 5:** Results of picking experiments.

Group	Number of fruits per cluster	Number of fruit clusters per group	Total number of fruits	Number of picking successes	Number of picking failures	Number of fruit damage	Picking success rate (%)	Fruit damage rate (%)	Average picking time per cluster (s)
1	3	5	15	15	0	0	100	0	7.3
2	4	5	20	20	0	0	100	0	7.8
3	5	5	25	22	3	2	88.0	9.1	9.3
4	6	5	30	25	5	3	83.3	12.0	11.6
5	7	5	35	28	7	3	80.0	10.7	12.5
	Total	25	125	110	15	8	88.0	7.3	9.7

According to **Table 5**, the average time for picking each cluster of fruits was 9.7 seconds, with a success rate of 88.0% and a damage rate of 7.3%. When the number of fruits picked in each cluster was 3~4, the picking success rate was 100%, and the fruit damage rate was 0. When the number of fruits picked in each cluster was 5~7, the more fruits picked in each cluster, the lower the picking success rate, and the higher the fruit damage rate.

**Figure 15:**
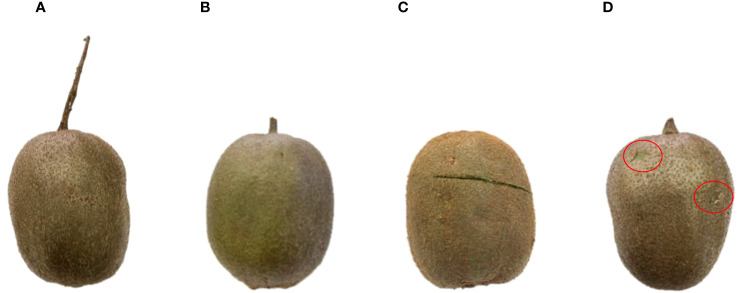
Status of picked fruits: **(A)** unpicked fruit from fruit trees, **(B)** undamaged picked fruits, **(C)** fruits damaged by the rotary cutter before all of the fruits entered the picking bin, **(D)** fruits with mutual collisions resulting in damage to the epidermis.

The causes of fruit damage were analyzed as follows: (**Figure 15A** represents the unpicked fruit from fruit trees; **Figure 15B** represents the undamaged picked fruits) ① Fruit leaves interfered with the color detection sensor, leading to damage to one or more fruits by the rotary cutter before they all entered the picking bin, as shown in **Figure 15C**. ② During picking or collection, fruit-to-fruit collisions could result in damage to the skin of overripe fruits (Note: Typically, when kiwifruit is picked in an orchard, the fruit is not fully ripe, and it is firmer, reducing the likelihood of skin damage), as shown in **Figure 15D**.

The factors contributing to fruit-picking failures were analyzed as follows. While kiwifruit grown on trellises typically fall within the same height range, the fruits become more irregular when a fruit cluster contains a higher number of fruits. Because the picking bin has a fixed size, some fruits may be blocked outside the picking bin during the process of enveloping fruits. However, the robot doesn’t adjust the enveloping angle correspondingly, leading to the failure to pick those fruits. Subsequently, there might be a second failure to pick the initially unpicked fruits within the cluster due to interference from branches or leaves.

## 7 Conclusion

1. For kiwifruit grown in orchard trellises, a multi-fruit picking kiwifruit robot was designed by analyzing kiwifruit cultivation parameters and design requirements. A continuous picking method of automatically recognizing fruits - fully enveloping fruits - non-destructively separating fruit stalks - and automatically collecting fruits was proposed, which can pick kiwifruits in clusters. The designed picking end-effector, after the fruit are recognized by sensors, uses envelopment to gather the fruits into the picking bin and then employs a rotary cutter mechanism to separate the fruit stalks without causing damage. Additionally, curved guide rods were also set to buffer and guide the picked fruit to reduce the rate of fruit damage.
2. The kinematic equations of a kiwifruit picking robot were established by the D-H method, and forward and inverse kinematic calculations were performed. Firstly, the robot reachable workspace and dexterous workspace ranges were solved using Robotics Toolbox in MATLAB software and the Monte Carlo method. Secondly, the trajectory planning of the robotic arm was performed by using the fifth-degree polynomial interpolation method, and the critical nodes in the picking path were calculated by using the RRT algorithm, furthermore, the scheme of the robot’s picking strategy was given. Finally, MATLAB was applied to simulate the motion trajectory of the kiwifruit picking robot to verify the feasibility of the trajectory planning scheme and the picking strategy.
3. A kiwifruit-picking test bed was established in a laboratory. A total of 125 fruits kiwifruits were selected and divided into five groups in the form of fruit clusters. The picking test was conducted with the mechanical arm set at a speed of 1.5m/s and the stepper motor running at 6r/min. The results showed that the average time required to pick each fruit cluster was 9.7 seconds, with a picking success rate of 88.0% and a picking damage rate of 7.3%. Moreover, the causes of fruit damage and fruit picking failures during the picking process were analyzed and elaborated in detail.
4. The research work of this study has a certain significance for the simultaneous harvesting of multiple fruits in kiwifruit, but it still needs to be further investigated in the future. For example, ① When the kiwifruit ripening season of the next year is approaching, picking experiments are conducted in real environments of kiwifruit orchards to test various index parameters of kiwifruit. ② Research on more advanced detection systems, on the one hand, can allow the robot to accurately locate the target fruit tree, on the other hand, can allow the end-effector to avoid the interference of external conditions such as fruit leaves, light, etc., to accurately locate the position of the target fruit. ③ The end-effector is further designed from the lightweight point of view on the premise of being able to achieve the target picking index.

## Data availability statement

The original contributions presented in the study are included in the article/supplementary material. Further inquiries can be directed to the corresponding author.

## Author contributions

MF: Conceptualization, Methodology, Validation, Writing – original draft. SG: Conceptualization, Formal analysis, Methodology, Software, Validation, Writing – original draft, Writing – review & editing. AC: Formal analysis, Supervision, Writing – review & editing. RC: Software, Writing – review & editing. XC: Supervision, Writing – review & editing.
